# Monitoring health-related quality of life in paediatric practice: development of an innovative web-based application

**DOI:** 10.1186/1471-2431-11-3

**Published:** 2011-01-12

**Authors:** Lotte Haverman, Vivian Engelen, Marion AJ van Rossum, Hugo SA Heymans, Martha A Grootenhuis

**Affiliations:** 1 Psychosocial Department, Emma Children's Hospital, Academic Medical Center, Meibergdreef 9, 1105 AZ Amsterdam, the Netherlands; 2 Paediatric Rheumatology, Emma Children's Hospital, Academic Medical Center, Meibergdreef 9, 1105 AZ Amsterdam, the Netherlands; 3 Paediatric Rheumatology, Jan van Breemen Instituut, Dr Jan van Breemenstraat 2, 1056 AB Amsterdam, the Netherlands

## Abstract

**Background:**

Health Related Quality of Life (HRQOL) questionnaires are increasingly used in clinical practice. These Patient Reported Outcomes (PROs) are provided to the paediatrician to facilitate communication with patients during a consultation. The aim of the current article is to describe the development and introduction of a new web-based application for the use of PROs in daily paediatric clinical practice.

**Methods:**

Currently, the use of PROs in daily clinical practice is very time consuming and often has logistical problems. The use of a web-based programme can overcome these problems and contributes to an improved use of PROs in clinical practice. We therefore developed an easily accessible website (KLIK) for outpatient treatment and a training programme for paediatricians to maximize the effectiveness and the practical use of PROs (KLIK PROfile).

**Results:**

The KLIK study was launched in August 2008 to evaluate the use of the KLIK PROfile in daily clinical practice. The KLIK study evaluates whether feedback from HRQOL data could influence patient satisfaction with the consultation, the advice given, the type of referrals and topics discussed. In this multicentre study, a control group (without the use of the KLIK PROfile) is compared to an intervention group (with the use of the KLIK PROfile). A sequential cohort design is chosen to avoid contamination between the study groups.

**Conclusions:**

Based on the positive experiences with the use of the KLIK PROfile acquired during the study we conclude that the KLIK PROfile may contribute to systematically monitor and discuss HRQOL issues during consultations. The next steps will be a comprehensive evaluation of the KLIK study data and the implementation of the KLIK PROfile in daily clinical practice in different patient groups.

## Background

The use of Patient Reported Outcomes (PROs) in daily clinical practice is receiving increasing attention. PROs include the self-assessment of functional status, symptoms or other concerns, such as patient needs and satisfaction with care. Health Related Quality of Life (HRQOL) questionnaires are commonly used in clinical trials to collect information about a specific group of patients. These questionnaires retrieve information directly from the patient and are therefore a form of PRO [1]. Today, HRQOL questionnaires are increasingly being used in daily clinical practice, being provided to the physician to facilitate communication with patients during a consultation. The majority of HRQOL studies focuses on oncology and reveals conflicting data in terms of effectiveness. Some adult studies suggest that discussing PROs improves communication between physicians and patients and facilitates early recognition of HRQOL problems [2-8]. Physicians generally consider the use of PROs as a valuable addition to daily health care [3]. Nevertheless, an improvement in patient satisfaction with care or an increase in scores is still challenging. This is due to methodological challenges, such as high baseline scores on patient satisfaction (ceiling effect [9-11]) or the nature of the study design. For example, randomization is not desirable because extra attention might be paid to HRQOL in all consultations (contamination).

In paediatrics, there is a particular need to address HRQOL in daily clinical practice. In the context of a child's development, the repeated measurement of HRQOL in different developmental stages can be valuable. The use of PROs allows HRQOL problems to be detected early and tailored intervention to be provided to the child before the HRQOL problems increase.

Children with a chronic disease are at a greater risk of HRQOL problems than their healthy peers [12]. In addition, for children compared to adults, it is difficult to respond spontaneously to their paediatrician's open questions during consultation. Therefore, a HRQOL questionnaire provided before the consultation might be useful to monitor, identify and discuss HRQOL issues faced by children with chronic illnesses. However, research into PROs in paediatrics is still scarce [13]. De Wit et al. showed that periodic monitoring and discussion of HRQOL in adolescents with diabetes improved their satisfaction with care. In addition, the use of PROs had a positive impact on their psychosocial wellbeing [14]. The aim of the current article is to describe the development and introduction of a new web-based application for use of PROs in daily paediatric clinical practice. Based on our experiences in paediatric oncology [15], we considered that 1) the need to address HRQOL issues, 2) the increased attention to PROs in clinical paediatrics and 3) the use of the internet in health care could be combined. In recent years, we have focused on the development of a new web-based application. In this article, we provide a detailed description of this process, the development of the website and a training programme for paediatricians to improve effectiveness in the use of PROs about HRQOL in paediatric practice and to make the use of HRQOL data more efficient.

## Methods

### The development of a web-based application: http://www.hetklikt.nu

Worldwide, only a few studies focus on the use of PROs in paediatrics. In the Netherlands we are currently performing two PRO studies in paediatrics: the QLIC-ON (Quality of Life in Childhood Oncology) study and the KLIK study (Dutch: Kwaliteit van leven in Kaart). The QLIC-ON study is aimed at child cancer patients in the period shortly after the end of successful treatment. In the QLIC-ON study much attention was paid to the presentation of the PROs and the development of a training programme for oncologists [15]. The results of the QLIC-ON study are promising, as they demonstrate that the monitoring of HRQOL increased discussion of emotional functioning and psychosocial functioning. Additionally, it improved the level of identification of emotional problems. Furthermore, the intervention does not lengthen consultation duration [16].

The KLIK study will be extensively described in this paper. Currently, HRQOL questionnaires are commonly completed at the clinic immediately before the actual consultation, with patients using stand-alone or touch screen computers. The results of these questionnaires are presented systematically. A printed version of this PRO (as applied in the QLIC-ON study) is handed to the physician to be discussed during the consultation. This method is very time consuming and often has logistical problems such as lack of privacy and room at the clinic [17,18].

The use of a web-based programme can overcome these problems and contributes to an improved use of PROs in clinical practice [19]. The website http://www.hetklikt.nu was developed based on the experiences of the QLIC-ON study. Children or their parents (depending on the age of the child) can complete the HRQOL questionnaires at home and paediatricians can retrieve these PROs directly from the website during the consultation.

### Use of the website

We organized the use of the web-based KLIK HRQOL application in our clinic as follows. Before the patient can complete the questionnaires, the researcher must enter the patient characteristics into the system. These include name, date of birth, email address, date of their visit and the name of the paediatrician. Based on date of birth the website selects the appropriate questionnaire for the child. Three days before the consultation, the website generates an automatic email asking the child or the parents to complete the HRQOL questionnaire. Two days after this first email, the website automatically generates a reminder email if the questionnaire has not yet been completed. In addition, the website is designed to be operational for different users; at present, the parents, the child and the paediatrician. Each user has a unique login name and is automatically given access to a specific secure section of the website.

**Figure 1:**
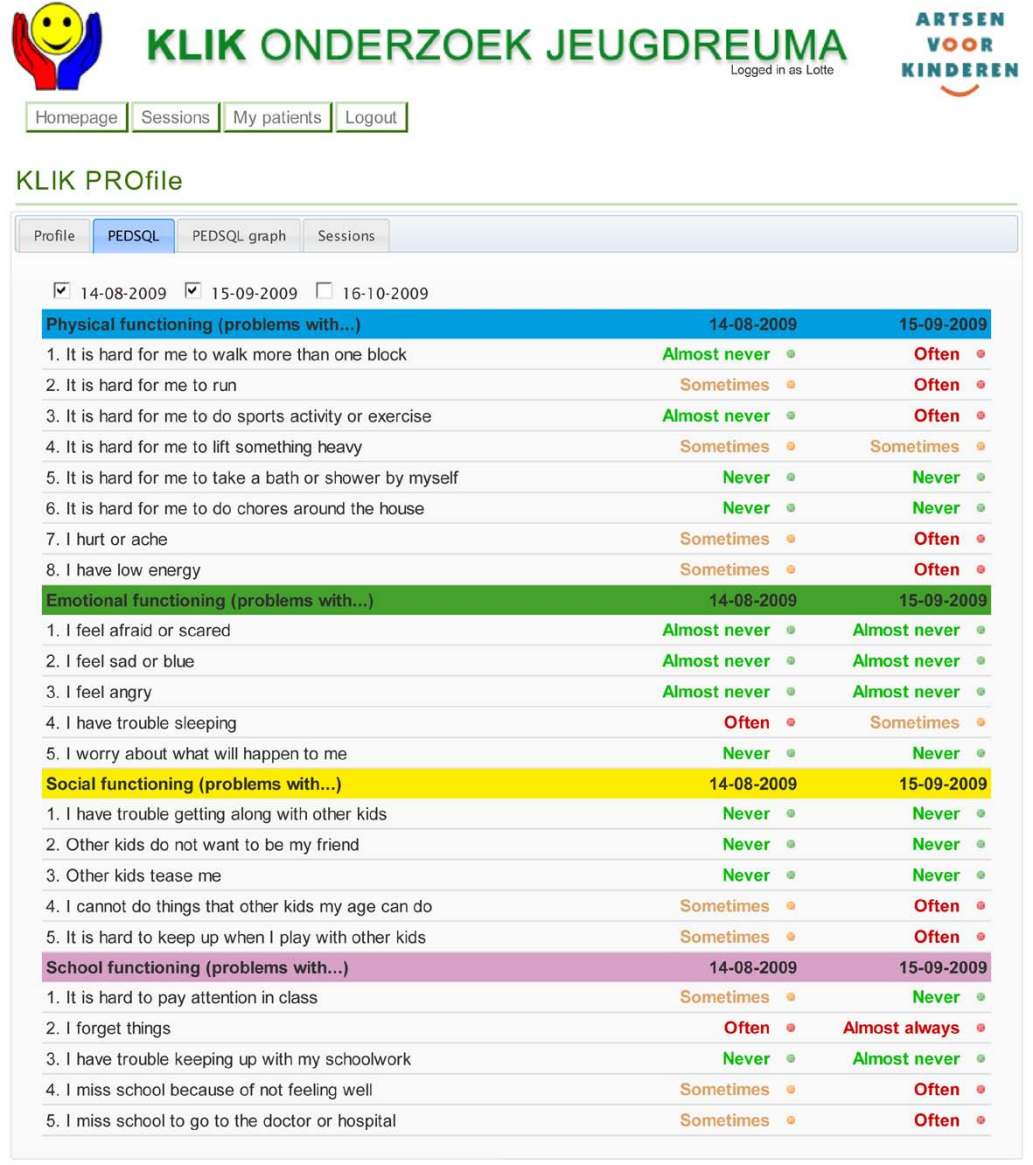
KLIK PROfile - A literal representation of the answers

**Figure 2:**
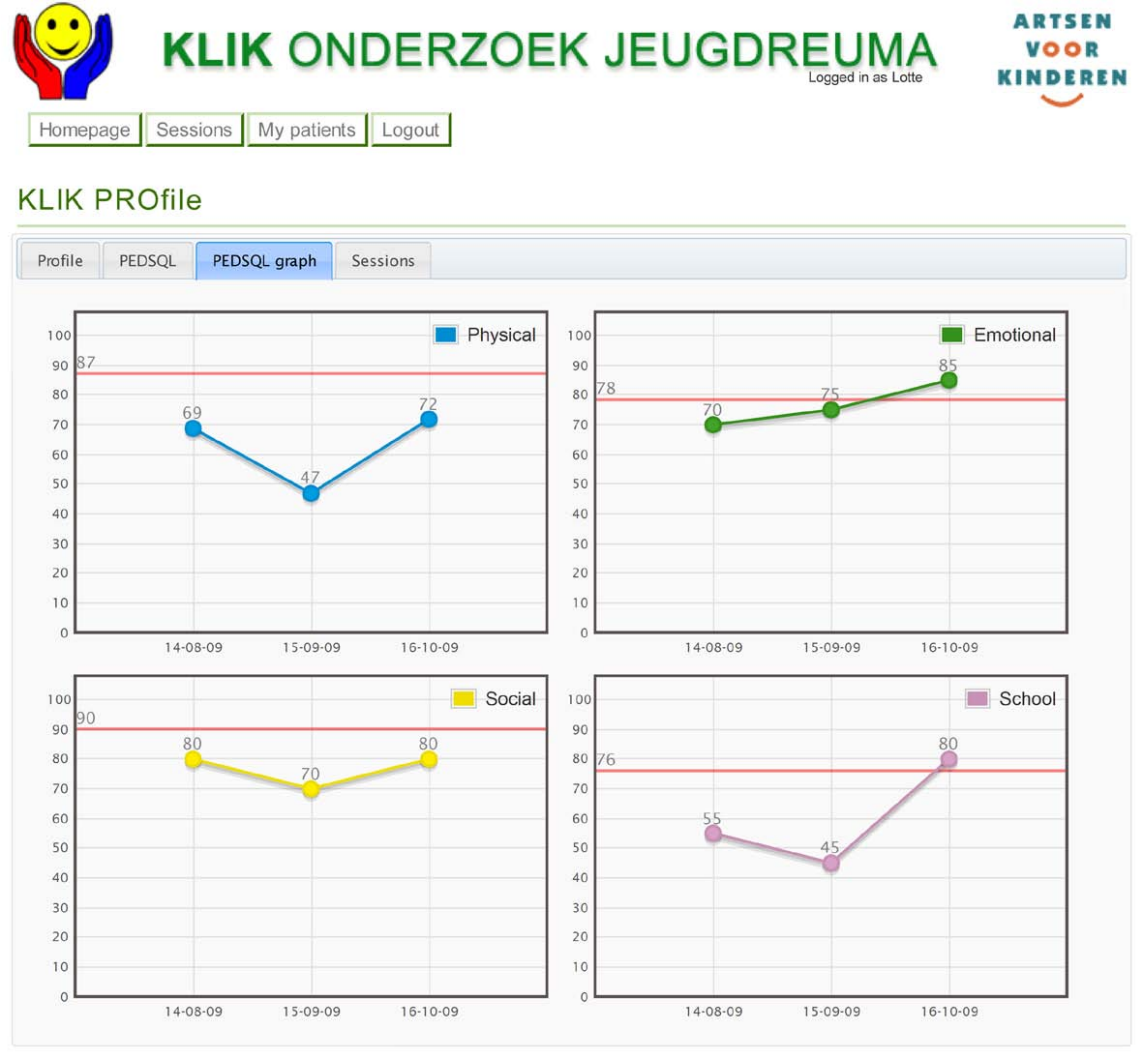
KLIK PROfile - Graphic presentation, including norm values

The website presents the answers of the child schematically in a so-called 'PROfile'. The development and implementation of the PROfile was based on the five conclusions presented by Greenhalgh et al. [15,20] which were also used with respect to the QLIC-ON PROfile. The PROfile consists of two parts. The first part is a literal representation of the answers on the item level. The answers are presented in red ('often', 'almost always') when a child reports experiencing problems regarding the subject, orange ('sometimes') when the child reports mild problems, or green ('never' and 'almost never') when a child reports not having problems. The second part consists of a graphic presentation of the PROs, including norm values. This makes it possible for the paediatrician to compare a child's HRQOL score to the healthy population norm. Moreover, longitudinal data can also be provided in the graphs, allowing the paediatrician to easily compare multiple HRQOL measurements for one child and detect profound changes over time [15]. An example of the PROfile is shown in Figures 1 and 2. The PROfile is visible to paediatricians as well as parents and the child, and it is printable and therefore can be added to the medical file. The use of the KLIK PROfile is illustrated by two cases (Additional file 1).

### Training

**Figure 3:**
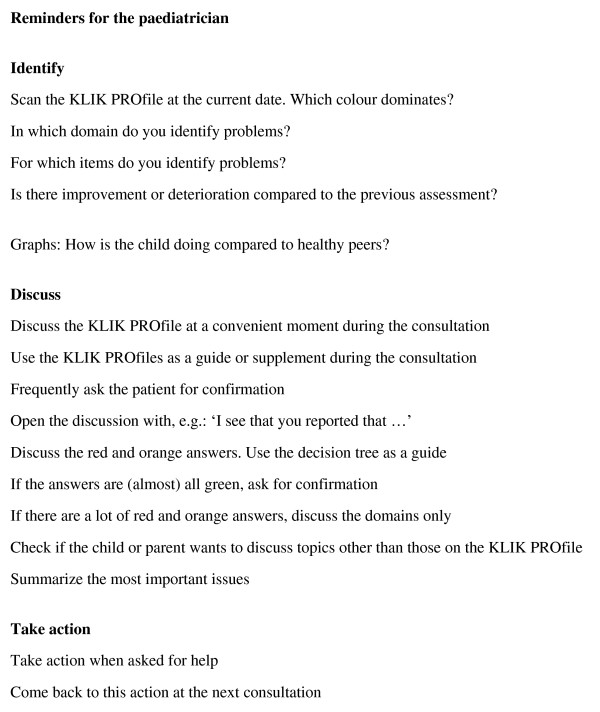
Reminders for paediatricians using the KLIK PROfile

To improve the use of PROs in clinical practice we invested in a training programme for paediatricians in the interpretation and use of the PROfile. The training course was given by a researcher and a psychologist, its duration was 90 minutes and it consisted of a short theoretical part and an extensive practical part. For the practical part, we used DVD material containing three short patient cases (duration: +/- 5 minutes), representing real consultations and actual PROfiles. Before the demonstration of each case, the KLIK PROfile was discussed ('How would you interpret and discuss this PROfile?') and the paediatricians received different assignments concerning each case. All of the cases had a specific learning goal, concerning either the general use of the KLIK PROfile, the use of line graphs or the use of a decision tree. After the demonstration of the cases, the skills of the paediatrician depicted on the DVD were evaluated and the paediatricians received a list of key reminders to assist in the use of the KLIK PROfile (Figure 3).

After the training session, the paediatricians received a pocket card, presenting a decision tree, an example of the KLIK PROfile and the reminders. We developed the decision tree in collaboration with paediatric psychologists, with the aim of supporting communication about the PROfile. The pocket card is also available on the website [15].

### Privacy

A major advantage of using the internet is that patients and parents are able to complete the questionnaires at home. Most importantly, children or parents can be invited at regular intervals to complete the questionnaires. Every patient is linked to their own paediatrician and the website only allows the paediatricians to see their own patients. Therefore, the privacy of the patients is guaranteed. Moreover, the website itself is designed to ensure the safety and protection of all data. We have secured the site with an SSL certificate and the information to and from the site is encrypted. In addition, the website server is positioned in a professional data centre and is physically secured against fire, forcible entry and vandalism. Professional web-builders are directly available for support of the website in the case of problems or emergencies. Within the scope of the KLIK study all data are stored anonymously. In addition, the database can easily be imported into Excel and SPSS to facilitate statistical analysis.

### Implementation

A web-based application also creates the opportunity for the KLIK PROfile to be used in different paediatric hospitals and facilitates collaboration between centres. Implementation of the KLIK PROfile is not limited to organizations or specific electronic medical records (EMR) of hospitals. Therefore, every paediatrician can easily use the KLIK website and PROfile.

## Results

### Study design and outcome measures of the KLIK study

Children and adolescents with Juvenile Idiopathic Arthritis (JIA) can experience problems in daily functioning, which may lead to a decreased HRQOL [21-23]. The KLIK study of these children was launched in August 2008 to evaluate the use of the KLIK PROfile in daily clinical practice. All patients (0 to 18 years) under treatment in one of the four paediatric rheumatology centres in Amsterdam were eligible for the KLIK study. To avoid contamination we chose to use a sequential cohort design. Randomization was not desirable because paediatricians had received training in use of the KLIK PROfile and it would not be possible for them to remain unbiased. Randomization of centres would also have introduced bias as the influence of the different care systems in the different centres would be too great.

Children and parents were allocated to the control group or intervention group depending on the date of the consultation. The control group (n = 79) was counselled between February 2009 and April 2009 and the intervention group (n = 121) between May 2009 and February 2010. The rheumatologists (n = 6) participated in both groups. Before the consultation, each child or their parents (if the child was younger than 8 years old) completed an HRQOL questionnaire at home using the KLIK website. For children aged 0-4 years, the TNO-AZL Preschool Children Quality of Life (TAPQOL) was used [24]. For children between 6-18 years, the Pediatric Quality of Life Scale (PedsQL) was applied [25]. The psychometric properties of both questionnaires have been proven satisfactory [24-26] and both questionnaires have a short completion time (5-10 minutes). The PedsQL appears to be the most appropriate HRQOL questionnaire because of its broad age range (6-18 years) and the availability of self-report as well as proxy report. In addition, Young et al. [26] have established the validity and reliability of the PedsQL online format for children with chronic health conditions.

In the control group, the KLIK PROfile was not provided to the paediatrician. In the intervention group, the PROfile was provided and discussed by the paediatrician during the consultation, with the focus on monitoring and discussing HRQOL problems. Shortly after the consultation, the child, the parents and the paediatrician completed a questionnaire, again using the website, about the topics discussed, referrals, advice, and satisfaction with the consultation. The intervention group also completed an evaluation of the PROfile. The study was approved by the Medical Ethics Committee of all participating centres. The outcome measures applied in the KLIK study were satisfaction with the consultation, advice, referrals and topics discussed. The outcomes of the intervention group will be compared to the control group, with the data collected to be analysed and published in 2011.

## Discussion

Only the results of the KLIK study can determine whether the online KLIK application has been effective. Nevertheless, here we can describe our first experiences with the KLIK application and make recommendations for future practice.

Since 2006 we have undertaken comprehensive research with the aim of realizing a web-based application that could systematically direct attention to HRQOL issues in daily paediatric clinical practice. The web-based PROfile appears to be an efficient application to achieve this goal.

In the context of using PROs in clinical practice, some issues should be considered. The use of the internet seems to be an efficient way to monitor HRQOL. However, the Netherlands has the highest rate of internet access in Europe. In 2009, 90 percent of all Dutch households had internet access [27]. Our experience with the KLIK study confirmed this. To accommodate the small number of patients without internet access at home an internet access point can be installed in the outpatient department. It is often assumed that completing the questionnaires and discussing HRQOL issues is very time consuming. However, the completion of the HRQOL questionnaire takes the child (or parents) no more than ten minutes [28]. In addition, several studies have demonstrated that discussing HRQOL issues does not increase the duration of the consultation [3].

In our opinion, to optimize the effect of the KLIK PROfile it is necessary to educate the paediatrician in its use. Due to lack of time, paediatricians are often not adequately trained, if at all, in the use of PROs [7,15,16]. Physicians are willing to discuss psychosocial problems with their patients but they often report that they consider themselves inadequately trained to discuss such health issues [29]. Thus, a training programme that focuses on discussing psychosocial aspects of chronic illness can be very valuable.

We incorporated the PedsQL questionnaire into the KLIK study. The PROfile questionnaire is used as a tool to monitor the domains of HRQOL which are important during childhood development. We emphasize that the PROfile cannot be used as a screening instrument to identify children at risk of psychosocial adjustment problems, but we are therefore extending the website with the Strengths and Difficulty Questionnaire [30,31] which can be used as a screening instrument.

The implementation of a web-based PROfile in clinical practice creates new challenges and opportunities. Depending on patient health care needs, members of a multidisciplinary team can use the KLIK PROfile individually or during a multidisciplinary consultation. In the future, the PROfile might be used not only by other practitioners (for example, psychologists or nurses), but also by different patient groups. The KLIK PROfile can be applied in all types of specialized and dedicated paediatric clinics. In addition, we assume that the PROfile can include both a generic part (such as the PedsQL) and a disease-specific questionnaire if available. The HRQOL scores can be compared to healthy norm scores or even to population-specific scores when enough data are available.

In addition to improving the care given to chronically ill children, we suggest that more attention should be paid to the needs of the parents. Children never visit a paediatrician on their own. The psychological mechanisms in paediatrics are diverse and complex and they influence the interaction between the parent, the child and the whole family system. For clinical practice, it is important not only to assess the illness of the child but also to evaluate the burden on the parents, their stress levels and reactions to the uncontrollable aspects of illness [32]. Parent Reported Outcomes could be an additional component in the implementation of PROs in the care of chronically ill children. Other studies have shown that parents can have major QOL problems related to the illness of their child [33].

## Conclusions

Based on the positive experiences with the use of the KLIK PROfile acquired during the study we conclude that the KLIK PROfile may contribute to systematically monitor and discuss HRQOL issues during consultations. The next steps will be the comprehensive evaluation of the KLIK study data and the implementation of the KLIK PROfile in daily clinical practice for different patient groups.

## Abbreviations

HRQOL: Health Related Quality of Life; PROs: Patient Reported Outcomes; JIA: Juvenile Idiopathic Arthritis; TAPQOL: TNO-AZL Preschool Children Quality of Life; PedsQL: Pediatric Quality of Life Scale; EMR: Electronic Medical Record

## Competing interests

There are no potential conflicts of interest arising from associations with commercial or corporate interests in connection with the work submitted.

## Authors' contributions

LH collected, analysed and interpreted the data and drafted the manuscript. MVR and MG designed and supervised the execution of the study, interpreted the data and drafted and revised the manuscript. VE and HH supervised the execution of the study and revised the manuscript. All authors have read and approved the final manuscript.

## Pre-publication history

The pre-publication history for this paper can be accessed here:

http://www.biomedcentral.com/1471-2431/11/3/prepub

## Supplementary Material

Additional file 1 **Two cases**. The use of the KLIK PROfile during consultation illustrated by two cases Click here for file
