# Supplementary material for: Monitoring health-related quality of life in paediatric practice: development of an innovative web-based application
Source: BMC Pediatr. 2011 Jan 12;11:3. doi: 10.1186/1471-2431-11-3 (PMC3025868; doi:10.1186/1471-2431-11-3)
Supplement: Additional file 1 — Two cases. The use of the KLIK PROfile during consultation illustrated by two cases [file 1471-2431-11-3-S1.DOC]

**Case 1**

Mary is an 11-year-old girl diagnosed with polyarticular juvenile idiopathic arthritis. On her first visit to the paediatric outpatient clinic, she had severe arthritis of several joints and was unable to go to school. She started anti-inflammatory treatment and slowly her joints improved. Mary frequently visited the outpatient clinic and became acquainted with her treating paediatric rheumatologist. Before the last two visits, she completed the HRQOL questionnaires online at home. Her PROfile was mainly green on the physical items, but she reported having problems with friends (social functioning) and sometimes feeling anxious and angry (emotional functioning). During the consultation her profile was discussed and her problems were addressed. Mary explained that she experienced a lack of understanding from her friends and that it made her angry. In addition, she was afraid of what would happen to her in the future. The paediatric rheumatologist advised her to give a presentation to her classmates about her disease and also explained the course of her disease to Mary once again. The paediatrician suggested to Mary and her mother that she would address these topics again during the next visit and if Mary did not feel any better a referral to the psychosocial department might be an option. At the end of this visit, Mary told her treating paediatric rheumatologist that she was relieved by the answers and advice, and she said she wished that she could use the KLIK PROfile for every visit because it made it much easier for her to talk to her doctor.

**Case 2**

Aaron is a 14-year-old boy diagnosed with systemic onset juvenile idiopathic arthritis (SoJIA). For nearly a year he was treated for SoJIA and achieved complete remission. His medication consisted of daily subcutaneous injections which he administered himself without ever complaining. Therefore, the treating paediatric rheumatologist did not expect Aaron to have HRQOL problems or problems with his treatment. The first time he used the questionnaire all the items on his PROfile were green, except for one. He indicated that he had huge problems with his medication, much to the surprise of the paediatrician. While discussing this Aaron explained that he was used to taking his medication at a precise time early in the morning, but during his summer holidays he had wanted to sleep in rather than get up early. Nevertheless, during the holidays he had dutifully set his alarm for early in the morning so he could take his medication, and never slept in. His adherence had not caused problems during the school term, so the paediatrician had little cause to ask him specifically about the timing of his medication and Aaron would not have discussed this issue himself without the use of the PROfile. Once the paediatrician knew about this problem it was easy to resolve by explaining to Aaron that he could take his medicine at a precise time a few hours later during his summer holidays.
